# Natural Variation of Hazelnut Allergenicity: Is There Any Potential for Selecting Hypoallergenic Varieties?

**DOI:** 10.3390/nu12072100

**Published:** 2020-07-16

**Authors:** Miguel Ribeiro, Joana Costa, Isabel Mafra, Sandra Cabo, Ana Paula Silva, Berta Gonçalves, Mélanie Hillion, Michel Hébraud, Gilberto Igrejas

**Affiliations:** 1 Department of Genetics and Biotechnology, University of Trás-os-Montes and Alto Douro, 5000-801 Vila Real, Portugal; jmribeiro@utad.pt; 2 Functional Genomics and Proteomics Unity, University of Trás-os-Montes and Alto Douro, 5000-801 Vila Real, Portugal; 3 LAQV-REQUIMTE, Faculty of Science and Technology, University Nova of Lisbon, 1000-001 Lisbon, Portugal; 4 REQUIMTE-LAQV, Faculdade de Farmácia, Universidade do Porto, Rua de Jorge Viterbo Ferreira, 228, 4050-313 Porto, Portugal; jbcosta@ff.up.pt (J.C.); isabel.mafra@ff.up.pt (I.M.); 5 Department of Biology and Environment, Centre for the Research and Technology of Agro-Environmental and Biological Sciences (CITAB), University of Trás-os-Montes and Alto Douro, 5000-801 Vila Real, Portugal; scsc90@hotmail.com (S.C.); bertag@utad.pt (B.G.); 6 Department of Agronomy, Centre for the Research and Technology of Agro-Environmental and Biological Sciences (CITAB), University of Trás-os-Montes and Alto Douro, 5000-801 Vila Real, Portugal; asilva@utad.pt; 7 INRAE, Plateforme d’Exploration du Métabolisme, composante protéomique (PFEMcp), 63122 Saint-Genès Champanelle, France; melanie.hillion@inrae.fr (M.H.); michel.hebraud@inrae.fr (M.H.); 8 Université Clermont Auvergne, INRAE, UMR0454 Microbiologie Environnement Digestif Santé (MEDiS), 63122 Saint-Genès Champanelle, France

**Keywords:** hazelnut allergy, genetic diversity, hazelnut-allergic patients’ sera, proteogenomics, immunoblot, nutrition

## Abstract

Hazelnuts (*Corylus avellana* L.) have an important role in human nutrition and health. However, they are a common cause of food allergy. Due to hazelnut varietal diversity, variety-dependent differences in the IgE-binding properties may be suspected, which could allow therapeutic strategies based on the use of hypoallergenic varieties to induce desensitization. In a proteogenomic approach, we aimed to evaluate the allergenic potential of a genetically diverse set of hazelnuts (n = 13 varieties). Minor differences were found at the level of genes encoding important allergens, namely Cor a 8, Cor a 9, and Cor a 14. Nevertheless, IgE-reactivity was similar for all varieties using sera from seven allergic individuals. The predominant IgE-reactive proteins were Cor a 9 (100%) and Cor a 1.04 (60%), with the former being the most frequently identified by a two-dimensional gel electrophoresis (2-DE)-based proteomic approach. Therefore, it seems that the conventional exclusion diet will hold its ground for the time being.

## 1. Introduction

Food allergy is increasingly prevalent in our societies and represents today an emerging public health problem affecting children, adolescents, and adults. This pathological reaction of the immune system is triggered by the exposure to a particular food protein in sensitized individuals and the clinical symptoms can range in severity from mild to life-threatening [1,2,3].

Hazelnut (*Corylus avellana* L.) is one of the most commonly consumed nuts worldwide, either raw or roasted, being largely used by the food industry in a variety of processed foods including cakes, creams, chocolates, and confectionary products. Moreover, hazelnuts are generally regarded as “heart-protective” foods and are considered important in human nutrition and health due to their protein, fatty acid, vitamins, essential minerals, essential amino acids, phenolics and dietary fiber composition [4]. Nevertheless, they are one of the most common triggers of IgE-mediated food allergies, being a major source of allergens capable of inducing mild to severe allergic reactions [5]. Hazelnut allergy is one of the most prevalent (7.2%) among global population, being particularly relevant in the Northern European countries due to its association with birch pollinosis. The USA presents the highest relative prevalence of sensitization to hazelnut (14.9%), followed by Germany (14.7%), Norway (12.8%), Switzerland (12.6%), and Sweden (11.8%) [6].

So far, twelve groups of allergenic proteins (Cor a 1, Cor a 2, Cor a 6, Cor a 8, Cor a 9, Cor a 10, Cor a 11, Cor a 12, Cor a 13, Cor a 14, Cor a 15 and Cor a TLP) were identified and characterized in hazelnut, all of them (except Cor a TLP) included in the International Union of Immunological Societies/World Health Organization (IUIS/WHO) official list of allergens (http://www.allergen.org/). From those, Cor a 8, Cor a 9, Cor a 11, and Cor a 14 have been associated with severe allergic reactions. Cor a 8 (9 kDa) and Cor a 14 (14–16 kDa) are small proteins of the prolamin superfamily, corresponding to non-specific lipid transfer proteins (nsLTP) and to 2S albumins, respectively. Cor a 8 and Cor a 14 are responsible for inducing moderate to severe/systemic clinical symptoms, being classified as major and minor allergens in hazelnut, respectively. Additionally, both proteins are highly stable to harsh food processing, thus preserving their allergenicity [4,7]. Cor a 9 (40 kDa/per subunit) and Cor a 11 (48 kDa/per subunit) are hexameric/trimeric storage proteins of the cupin superfamily, corresponding to the 11S legumin-type globulins and 7S vicilin-type globulins, respectively. Like Cor a 8, they are classified as major allergens, provoking severe and systemic allergic reactions. Cor a 9 and Cor a 11 are thermostable proteins, which can maintain their allergenic potential after common food processing [8].

The standard of care of hazelnut allergy and other food allergies is not optimal because it relies on the exclusion of the allergenic foods from patients’ diets and the allergen-induced systemic reactions are treated with adrenaline [1,3]. However, depending on the intensity of the allergic reactions and considering the novel immunotherapies (administration of small amounts of hazelnut to induce desensitization), the selection of hazelnut varieties with less allergen expression might be preferred for this type of strategies. Individuals with minor hazelnut-induced allergic reactions might also consider carefully selecting specific hazelnut varieties for possible consumption. In fact, hazelnut is a wind-pollinated species and it has a self-incompatible mating system that enforces cross-pollination; thus, genetic diversity is expected to be high in naturally occurring plants [9]. The information on overall gene transcription patterns of hazelnut allergens is still very scarce [10,11] and their correlation with expressed proteins/allergens and its allergenic potential was not described. In this context, this project intends to investigate how natural inter-varietal genetic differences can affect the expression level of hazelnut allergens and their allergenic potential, aiming at selecting genotype candidates more suited for the consumer’s health, as the basis of promising desensitization strategies.

## 2. Materials and Methods

### 2.1. Plant Material

Thirteen hazelnut (*Corylus avellana* L.) genotypes were studied. Fruits were collected from trees in full production in two hazelnut orchards located in Moimenta da Beira (lat. 40°59′ N; long. 7°37′ W, 644m a.s.l.) and Vila Real (lat. 40°59′ N; long. 7°37′ W, 644m a.s.l.) in the north of Portugal, during 2016: Grada de Viseu (Moimenta da Beira, Portugal), Butler, Cosford, Ennis, Fertile de Coutard, Grossal, Longue d’Espagne, Lansing, Merveille de Bollwiller, Morell, Negreta, Segorbe and Tonda di Giffoni (Vila Real, Portugal). After that, the shells were broken and the kernels were milled with a commercial blender (Taurus, Barcelona, Spain) into a fine powder. Hazelnut flours were then defatted by means of several passages in acetone, followed by paper filtration, and then dried overnight as previously described [12].

### 2.2. DNA Extraction

DNA was extracted from 100 mg of defatted hazelnuts using the commercial Nucleospin Food kit (Macherey-Nagel, Düren, Germany), according to the manufacturer’s instructions with minor alterations, which included the incubation with 2 μL of RNase A (2 mg/mL) at room temperature for 5 min, after the lysis step. Yield and purity of the DNA extracts were assessed by UV spectrophotometric DNA quantification on a Synergy HT multi-mode microplate reader (BioTek Instruments, Inc., Vermont, USA), using a Take3 micro-volume plate accessory and using the nucleic acid quantification protocol with sample type defined for double-strand DNA in the Gen5 data analysis software version 2.01 (BioTek Instruments, Inc., Vermont, USA). The integrity of DNA was evaluated by electrophoresis in a 1.0% agarose gel containing 1× Gel Red (Biotium, Hayward, CA, USA) for staining and carried out in 1× STGB (GRISP, Porto, Portugal) for 25 min at 200 V. Using the UV light tray Gel Doc™ EZ System (Bio-Rad Laboratories, Hercules, CA, USA), a digital image of the agarose gel was obtained with Image Lab software version 5.2.1 (Bio-Rad Laboratories, Hercules, CA, USA). All the extracts were kept at −20 °C until further analysis.

### 2.3. Gene Selection and Primer Design

Sequences from three genes encoding specific hazelnut allergens, namely the Cor a 8, Cor a 9 and Cor a 14 with accession numbers AF329829.1, AF449424.1 and FJ358504.15, respectively, were retrieved from NCBI database. In silico analysis was performed for each sequence in order to determine the region with more specificity (without cross-reactivity with other species). Two sets of specific primers were designed for each sequence, one for real-time PCR amplification and one for sequencing purposes (Table S1, supplementary material). All primers were submitted to a basic local alignment search tool BLAST (http://blast.ncbi.nlm.nih.gov/Blast.cgi) to test their in-silico specificity and to OligoCalc (http://www.basic.northwestern.edu./biotools/oligocalc.html) to evaluate their properties, self-hybridization and the absence of hairpins. The oligonucleotides were synthesized by Eurofins Genomics Germany GmbH (Ebersberg, Germany).

### 2.4. Real-Time PCR

Real-time PCR assays were carried out in 20 μL of total reaction volume, containing 2 μL of DNA (20 ng), 1 × SsoFast EvaGreen Supermix (Bio-Rad Laboratories, Hercules, CA, USA) and 350 nmol/L of each primer Cor 8F/Cor 8R, Cor 9F/Cor 9F or Cor 14F/Cor 14R (Table S1, supplementary material). The assays were performed in a fluorometric thermal cycler CFX96 Real-time PCR Detection System (Bio-Rad Laboratories, Hercules, CA, USA), according to the following protocol: 95 °C for 5 min, 50 cycles at 95 °C for 15 s and 65 °C for 40 s, with the collection of fluorescence signal at the end of each cycle. In order to confirm the amplified PCR products, the real-time PCR program included a melting curve analysis. PCR products were denatured at 95 °C for 1 min and then annealed at 65 °C for 3 min (for the correct formation of DNA duplexes), being followed by a melting curve (65 °C up to 90 °C) with temperature increments of 0.2 °C every 10 s. The fluorescence data were acquired at the end of each melting temperature, being analyzed by the software Bio-Rad CFX Manager 3.1 (Bio-Rad Laboratories, Hercules, CA, USA). The real-time PCR trials were performed for each gene, using four replicates for each hazelnut variety.

### 2.5. Sequencing

The identity of amplified PCR products by real-time PCR was confirmed by sequencing. Considering that the real-time PCR amplicons are rather small for direct sequencing (110–169 bp), other primer sets encompassing the target regions were specifically designed to produce larger fragments (339–417 bp) (Cor 8FS/Cor 8RS, Cor 9FS/Cor 9RS and Cor 14FS/Cor 14RS) (Table S1, supplementary material). The sequencing primer sets were used to amplify the 13 hazelnut varieties by end-point PCR, as described in Section 2.6. The PCR products were purified with GRS PCR & Gel Band Purification Kit (GRISP, Porto, Portugal) to remove interfering components and sent to a specialized research facility for sequencing (Eurofins Genomics Germany GmbH, Ebersberg, Germany). Each PCR product was sequenced twice, considering the dual coverage of the target fragment with direct sequencing of both strands in opposite directions, enabling the production of two high quality complementary sequences. The quality of electropherograms was evaluated using the FinchTV software (Geospiza, Seattle, WA, USA) and the sequences were further aligned with the software BioEdit v7.2.5 (Ibis Biosciences, Carlsbad, CA, USA).

### 2.6. End-Point PCR

PCR assays were performed for sequencing purposes, using a total reaction volume of 25 μL, containing 2 μL of DNA extract (20 ng), 67 mmol/L of Tris-HCl (pH 8.8), 16 mmol/L of (NH_4_)_2_SO_4_, 0.01% of Tween 20, 200 μmol/L of each dNTP, 1.0 U of SuperHot Taq DNA Polymerase (Genaxxon Bioscience, Ulm, Germany), 3.0 mmol/L of MgCl2 and 280 nmol/L of each primer (Cor 8FS/Cor 8RS, Cor 9 FS/Cor 9RS or Cor 14FS/Cor 14RS) (Table S1, Supplementary Material). The assays were carried out in a MJ Mini™ Gradient Thermal Cycler (Bio-Rad Laboratories, Hercules, CA, USA), using the same temperature program with the three primer sets: initial denaturation at 95 °C for 5 min, 40 cycles at 95 °C for 30 s, 63 °C for 45 s, 72 °C for 60 s and a final extension at 72 °C for 5 min. The PCR products were analyzed by electrophoresis as described in Section 2.2., using 1.5% agarose gels.

### 2.7. Protein Extraction

Hazelnut proteins were extracted as described by Pastorello, Vieths, Pravettoni, Farioli, Trambaioli, Fortunato, Luttkopf, Calamari, Ansaloni, Scibilia, Ballmer-Weber, Poulsen, Wutrich, Hansen, Robino, Ortolani and Conti [12]. Briefly, defatted hazelnut samples were suspended (1:10, *w*/*v*) in 0.1 mol/L phosphate buffered saline (PBS, pH 7.4). The mixture was incubated at 4 ℃ with stirring for 2 h. Then, the mixture was centrifuged at 30,000× *g* for 20 min at 4 ℃ and the supernatant collected. In the case of two-dimensional electrophoresis (2-DE), defatted hazelnut samples were suspended (1:10, *w*/*v*) in extraction solution containing 4% (*w*/*v*) CHAPS (7 mol/L urea, 2 mol/L thiourea, 1% (*v*/*v*) immobilized pH gradient (IPG) buffer), 20 mmol/L of DTT in milliQ ultrapure water, as described elsewhere [13]. The mixture was stirred for 1 h at 4 ℃, disrupted by sonication with an ultrasonic homogenizer (Vibra-CellTM VCX130, Sonics & Materials Inc., Newtown, CT, USA) in three bursts of 10 s at 30% of full power [14], centrifuged at 30,000× *g* for 20 min at 4 ℃ and the supernatant collected. Protein concentration was in both cases determined using the 2D Quant kit (GE Healthcare, Buckinghamshire, UK) following the manufacturer’s instructions.

### 2.8. One-Dimensional Electrophoresis (1-DE)

The hazelnut protein extracts were separated by electrophoresis using two different systems: a big gel system (160 × 180 × 1 mm) in order to obtain maximum protein separation for profiling and band analysis, and a small gel system (80 × 80 × 1 mm) to run immunoblotting with the sera of hazelnut-allergic individuals. Briefly, concerning larger gels, hazelnut proteins were resuspended in sample buffer (2% SDS, 40% glycerol, 0.02% bromophenol blue, 0.08 M Tris-HCl pH 8.0) and separated in a resolving gel using 12.52% T (total monomer concentration) and 0.97% C (weight percentage of crosslinker) and tris-glycine buffer. When reducing conditions were applied, to the sample buffer was added 1% (*w*/*v*) dithiothreitol (DTT). The gels were stained with Coomassie Blue R-250 for 24 h and then washed in distilled water overnight [15]. Coomassie-stained gels were scanned with a flatbed scanner (Umax PowerLook 1100, Fremont, CA, USA). The digital images were analyzed with CLIQS 1D Pro software (TotalLab, UK), which detects bands and generates a binary matrix reflecting the presence/absence of specific bands. For small gels, proteins were resuspended in Bolt™ LDS sample buffer with 10% (*v*/*v*) Bolt™ Sample Reducing Agent (Thermo Fisher Scientific, Waltham, MA, USA) and separated in precast Bolt Bis-Tris Plus gels using 4–12% gradient polyacrylamide concentration and MES SDS Running Buffer (Thermo Fisher Scientific, Waltham, MA, USA). The gels were stained as above-mentioned or used for immunoblotting assays.

### 2.9. Two-Dimensional Electrophoresis (2-DE)

2DE sample extracts were first resuspended in a rehydration solution, consisting of an extraction solution with bromophenol blue up to the volume recommended by the manufacturer for the immobilized pH (3–10) gradient (IPG) strips (7 cm) [13,16]. Isoelectric focusing (IEF) of 80 µg of protein was performed on an Ettan™ IPGPhor II™ system (Amersham Biosciences, Uppsala, Sweden) using the following conditions: a first step of active rehydration was performed at 50 V for 12 h, followed by a linear gradient up to 300 V for 30 min; a step at 300 V for 30 min; a linear gradient up to 1000 V for 30 min; a linear gradient up to 5000 V for 1.5 h and finally, a step at 5000 V for 45 min. Focused IPG strips were equilibrated twice for 15 min in equilibration buffer [(6 mol/L urea, 30% (*w*/*v*) glycerol, 2% (*w*/*v*) sodium dodecyl sulphate (SDS) in 0.05 mol/L Tris–HCl buffer pH 8.8)]. In the first equilibration step, 1% DTT was added to the original equilibration buffer, and 4% iodoacetamide to the second step. Bromophenol blue was added to both solutions. The equilibrated IPG strips were gently rinsed with MES electrophoresis buffer, softly dried to remove excessive buffer, and then applied to NuPAGE™ 4–12% Bis-Tris ZOOM™ Protein Gel (Thermo Fisher Scientific; 80 × 80 × 1 mm; 4–12%; IPG-well). After electrophoresis, the 2-D gels were used for immunoblotting or fixed in 10% (*v*/*v*) acetic acid, 40% (*v*/*v*) methanol solution for 1 h, followed by Coomassie Brilliant Blue G-250 staining overnight. Excess staining was removed by rinsing the gels with 20% (*v*/*v*) methanol solution. Coomassie-stained gels were scanned with a flatbed scanner (Umax PowerLook 1100; Fremont, CA, USA) and the digitized images were analyzed using Lab Scanner Image Master 5.0 software (Amersham Biosciences; GE Healthcare) and Progenesis SameSpots v4.5 (Non-linear Dynamics Limited, Newcastle, UK).

### 2.10. Allergic Patients and Immunoblot

Sera were obtained from hazelnut-allergic subjects from Centro Hospitalar de Trás-os-Montes and Alto Douro (CHTMAD) and PlasmaLab International (Everett, MA, USA). Table 1 shows the clinical features of the allergic individuals enrolled in this study.

Hazelnut specific IgE was quantified with the ImmunoCAP system using complete hazelnut allergen f17 (Phadia, Uppsala, Sweden). All sera were stored at −80 °C until analysis. The experiments were approved by the ethical committee of CHTMAD. Both 1-DE and 2-DE gels were electroblotted onto nitrocellulose membrane using an iBlot 2 Dry Blotting System (Invitrogen, Carlsbad, CA, USA): 20 V for 1 min, 23 V for 4 min and 25 V for 5 min. The membrane was blocked with 5% dry milk in 1 × TBST (0.01M Tris-Based Saline with 0.1% Tween 20) for 1 h, incubated with the sera of allergic patients or negative controls (overnight at 4 °C with agitation) and then with the anti-human IgE (ε-chain specific)—peroxidase antibody (Sigma Aldrich, St. Louis, MO, USA) for 1 h at room temperature. Between incubation steps, the membranes were always washed 3 times with 1 × TBST.

For 2-DE immunoblot, pooled sera of the hazelnut-allergic individuals were used. Negative controls were used both for 1-DE and 2-DE immunoblotting and consisted in the incubation using the sera of non-allergic individuals; no IgE reactivity was observed (Figure S1, Supplementary Material). Several combinations of hazelnut protein amounts and serum dilutions (in TBST) were tested to avoid clotting and to minimize the non-specific antibody response. The final conditions were 40 µg of protein per lane for 1-DE and 80 µg of protein for 2-DE with a serum dilution of 1:10. The results were visualized with chemiluminescent horseradish peroxidase (HRP) substrate (WesternBright™ ECL kit, Advansta Inc., San Jose, CA, USA) and X-ray films (LucentBlue X-ray film, Advansta Inc., San Jose, CA, USA). Densitometric analysis was conducted using reversible Ponceau staining as loading control [17]. Immunoblot analyses were performed for all the hazelnut varieties using the sera of hazelnut-allergic patients, individually (1-DE) or in pooled sera (2-DE), in two different experiments (each with two replicates).

### 2.11. Mass Spectrometry and Protein Identification

Selected immunoreactive 2-DE gel pieces (spots) were initially distained with 25 mmol/L ammonium bicarbonate in 5% acetonitrile for 15 min, followed by two steps with 25 mmol/L ammonium bicarbonate in 50% acetonitrile for 30 min each. Then, spots were dehydrated with 100% acetonitrile for 10 min. In order to completely remove the solvent, they were dried in a speedvac for about 10 min. For protein digestion, 100 ng of trypsin (Promega, Wisconsin, USA) in 25 mmol/L ammonium bicarbonate were added to each spot. Samples were further incubated at 37 °C overnight. Peptides were extracted from the gel pieces by the addition of 100% acetonitrile to the solution used for digestion (final concentration of acetonitrile around 45–50%) and sonication for 10 min. Prior to nanoscale liquid chromatography coupled to tandem mass spectrometry (nano LC-MS/MS) analysis, the samples were dried in a speedvac for around 30–45 min. Finally, the samples were dissolved in 10 µL of trifluoroacetic acid (TFA) 0.05% by sonication for 10 min and transferred into vials. A sample injection volume of 6 µL, a pre-column (C18 PepMap300, 5 µm, 300 A, 300 µm × 5 mm) and a separation column (Acclaim PepMap 100, C18, 75 µm × 25 cm nanoViper) were used for a gradient elution with eluents A (0.1% formic acid, 5% dimethyl sulfoxide (DMSO)) and B (0.1% formic acid, 5% DMSO in acetonitrile), following the program: 0 min 4% B, 5.1 min 4% B, 20 min 40% B, 21 min 90% B, 26 min 90% B, 26.1 min 4% B, 30 min 4% B, flow rate at 0.3 µL/min.

Mass spectrometry analysis was performed using a LTQ-Orbitrap Velos (Thermo Fisher Scientific, Waltham, MA, USA) mass spectrometer, operated in positive ion mode. MS scans were acquired with resolution of 15,000. Mass range: 375–1400 m/z. MS^2^ scans were acquired in Fourier transform mass spectrometry (FTMS) with resolution of 7500. For protein identification, a database search was performed using Mascot Daemon (v. 2.5.1) software and Mascot search algorithm (Matrix Science Inc., Boston, MA, USA). MS and MS/MS spectra were searched against a database containing 476 *Corylus avellana* L. protein sequences retrieved from UniProt (https://www.uniprot.org/). Search parameters were set as follows: tryptic specificity with maximum of 2 miscleavages, cysteine carbamidomethylation (C), methionine oxidation (M) and deamidation (NQ) as variable modifications, precursor mass tolerance of 20 ppm, fragment mass tolerance of 0.05 Da, false discovery rate of 1%. Spectra were also searched against a contaminant database. An identification was validated when more than four unique peptides were found.

### 2.12. Statistical Analysis

The binary matrix produced by the CLIQS 1D Pro software (TotalLab, Newcastle upon Tyne, UK) was used for genetic analysis using GenAlEx 6.5 software (Australian National University, Canberra, Australia) [18], which allowed calculating the diversity of the set under study. An unweighted pair group method with arithmetical averages (UPGMA) dendrogram of genetic similarity was generated using the Jaccard’s similarity coefficient in NTSYS software (Exeter software, Setauket, NY, USA), v. 2.1 [19]. The adjustment of the UPGMA dendrogram to the binary matrix was evaluated by the cophenetic matrix correlation with COPH and MXCOMP modules of NTSYS software. The results are expressed as mean ± standard deviation (SD) unless otherwise stated. Differences among the different samples were determined by one-way analysis of variance (ANOVA). Multiple comparisons were performed using Tukey’s post-hoc test, and the criterion for significance was *p* < 0.05 (GraphPad Prism v6.03, GraphPad Software, La Jolla, CA, USA).

## 3. Results and Discussion

### 3.1. Cor a 8, Cor a 9 and Cor a 14 Gene Quantification

**Figure 1:**
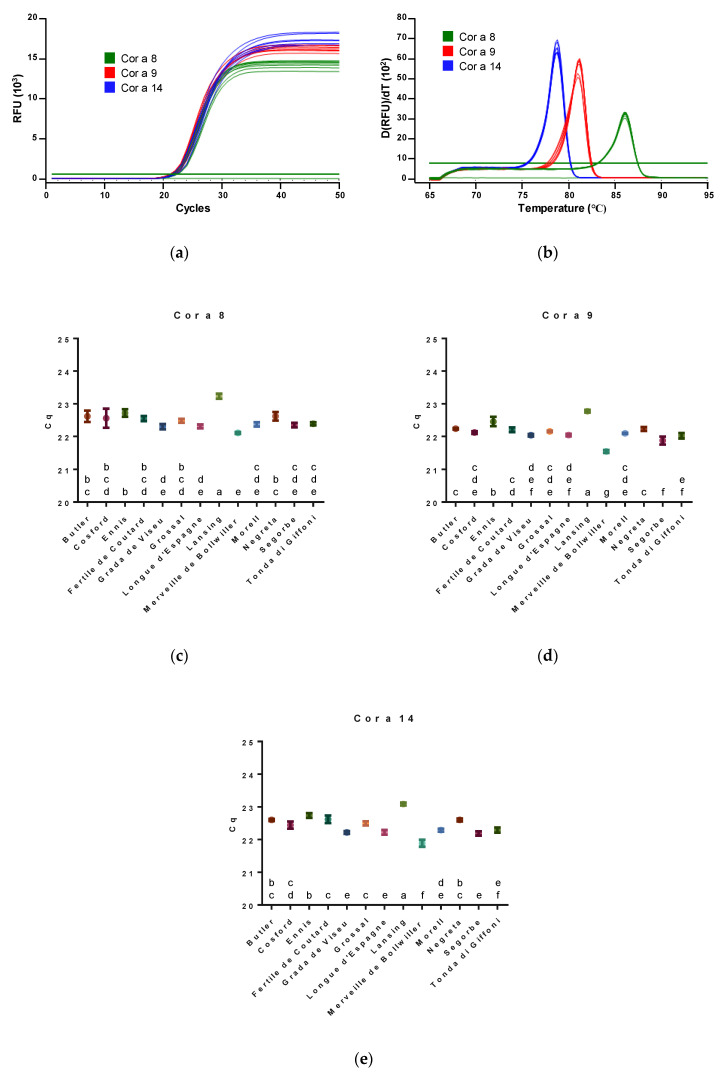
(**a**) Amplification curves and (**b**) melting peaks of different hazelnut varieties analyzed by real-time PCR using EvaGreen dye. Melting temperatures of 78.8 °C, 81.2 °C and 86.0 °C for PCR products from Cor a 14, Cor a 9 and Cor a 8, respectively. (**c**) Cor a 8, (**d**) Cor a 9 and (**e**) Cor a 14 cycles of quantification (Cq) values for the amplification of 20 ng of DNA from the different hazelnut varieties. Values with the same letter (a, b, c, d, e, f, g) do not differ significantly at *p* < 0.05.

At the genomic level, our main objective was to assess the amplification profile of genes encoding allergens involved in severe allergic responses, such as Cor a 8, Cor a 9 and Cor a 14 [4,7], and to verify variety-dependent differences as a starting point for subsequent analyzes based on proteomics. DNA regions of genes encoding different hazelnut allergens were selected from NCBI database and specific primers were designed in order to produce amplicons with 118 bp, 169 bp and 122 bp for Cor a 8, Cor a 9 and Cor a 14 genes, respectively. Real-time PCR assays using EvaGreen dye were carried out targeting the selected genes. Melting curve analysis confirmed the identity of PCR products, enabling the amplification of products with distinct melting peaks, namely at 78.8 °C, 81.2 °C and 86.0 °C for Cor a 14, Cor a 9 and Cor a 8 genes, respectively (Figure 1a,b).

In order to eliminate potential differences caused by primer mismatches in the annealing regions, all varieties were sequenced using broader regions that encompassed the real-time PCR products of each target gene (Figure S2–S4, Supplementary Material). In all three genes, the target sequences amplified by real-time PCR had no mismatches with the consensus sequences where primers were designed. Additionally, within the same target regions there were no mismatches among distinct varieties (Figure S2–S4, Supplementary Material).

The amplification curves showed similar profiles within hazelnut varieties and studied genes, with cycles of quantification (Cq) values ranging from 22 to 23 cycles (Figure 1c–e). In general, all hazelnut varieties presented lower Cq values for Cor a 9 gene (Figure 1d), which was expected since Cor a 9 is a major protein in hazelnut. Cq values for Cor a 8 and Cor a 14 encoding genes were more similar among varieties, which is in good agreement with the fact that they are both minor hazelnut proteins [4] (Figure 1c,e). Nevertheless, some differences can be verified among distinct varieties and among genes. Considering the Cor a 8 Cq values, the relationships are as follows: ‘Lansing’ > ‘Ennis’ ≥ ‘Butler’ = ‘Negreta’ ≥ ‘Cosford’ = ‘Fertile de Coutard’ = ‘Grossal’ ≥ ‘Tonda di Giffoni’ = ‘Morell’ = ‘Segorbe’ ≥ ‘Longue d’Espagne’ = ‘Grada de Viseu’ ≥ ‘Merveille de Bollwiller’. For Cor a 9: ‘Lansing’ > ‘Ennis’ > ‘Butler’ = ‘Negreta’ ≥ ‘Fertile de Coutard’ ≥ ‘Grossal’ = ‘Cosford’ = ‘Morell’ ≥ ‘Longue d’Espagne’ = ‘Grada de Viseu’ ≥ ‘Tonda di Giffoni’ ≥ ‘Segorbe’ > ‘Merveille de Bollwiller’. Finally, Cor a 14 presented the following results: ‘Lansing’ > ‘Ennis’ ≥ ‘Negreta’ = ‘Butler’ ≥ ‘Grossal’ = ‘Fertile de Coutard’ ≥ ‘Cosford’ ≥ ‘Morell’ = ‘Tonda di Giffoni’ ≥ ‘Longue d’Espagne’ = ‘Grada de Viseu’ = ‘Segorbe’ > ‘Merveille de Bollwiller’. These results clearly show that despite the similarity among amplification profiles within each target gene, the varieties ‘Lansing’ and ‘Merveille de Bollwiller’ consistently present the highest and lowest Cq values, respectively. Therefore, a higher allergenic potential for the ‘Merveille de Bollwiller’ could be expected. In fact, Garino, Locatelli, Coïsson, D’Andrea, Cereti, Travaglia and Arlorio [10] investigated the differential transcript abundance of hazelnut allergens Cor a 1, Cor a 8 and Cor a 11 encoding genes through real-time PCR and they found that the transcription level of Cor a 1 gene did not vary among the studied varieties, whereas Cor a 8 and Cor a 11 showed a different inter-varietal transcription level, achieving a maximum eightfold difference for the Cor a 11 gene between ‘Tonda Romana’ and ‘Palaz’. Still, these results must be carefully interpreted; for example, it is difficult to classify whether the variation in amplification results among Cor a 8 (Cq values of 22.11–23.23), Cor a 9 (Cq values of 21.54–22.78), and Cor a 14 (Cq values of 21.89–23.09) genes, has actually any biological significance. In this sense, proteomics will provide an important link to genomic data and functional biology [20,21], gathering information about changes in protein expression and potential allergenicity of the different hazelnut varieties analyzed.

### 3.2. Protein Polymorphism

**Figure 2:**
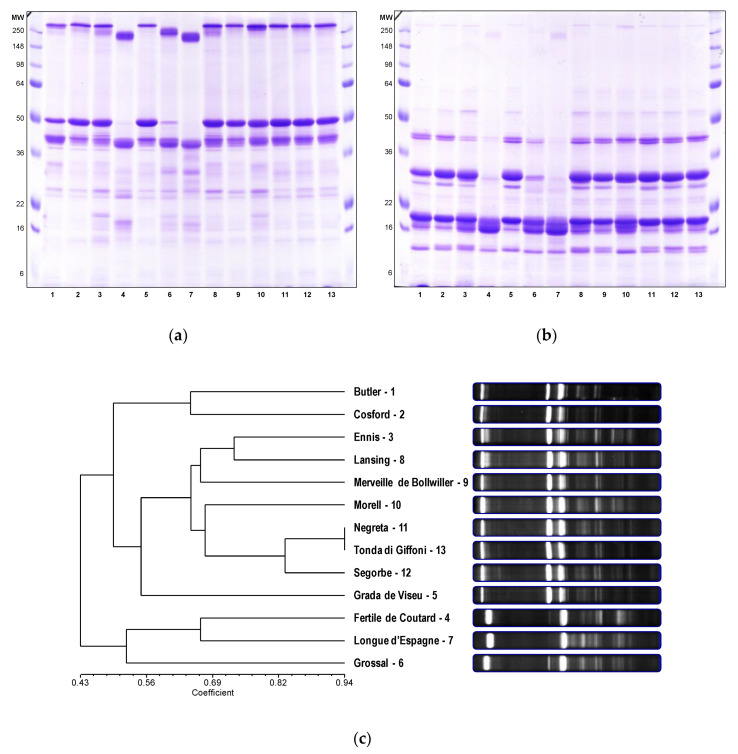
(**a**) Native and (**b**) reduced hazelnut protein electrophoretic profiles analyzed by SDS-PAGE using a 12% gel. Legend: lane 1, ‘Butler’; lane 2, ‘Cosford’; lane 3, ‘Ennis’; lane 4, ‘Fertile de Coutard’; lane 5, ‘Grada de Viseu’; lane 6, ‘Grossal’; lane 7, ‘Longue d’Espagne’; lane 8, ‘Lansing’; lane 9, ‘Merveille de Bollwiller’; lane 10, ‘Morell’; lane 11, ‘Negreta’; lane 12, ‘Segorbe’; lane 13, ‘Tonda di Giffoni’. Sizes (in kDa) of the protein molecular weight marker (SeeBlue™ Plus2 Pre-stained Protein Standard, Thermo Fisher Scientific, Massachusetts, USA) are shown on the left and right of the gel. (**c**) Dendrogram of genetic similarity among the 13 hazelnut varieties based on protein markers. The unweighted pair group method with arithmetic mean was calculated with the Jaccard’s similarity coefficient. The cophenetic correlation coefficient (r) for this matrix was 0.846.

Hazelnut proteins were analyzed using a large (160 × 180 × 1 mm) sodium dodecyl sulfate polyacrylamide gel electrophoresis (SDS-PAGE) system to allow better resolution and band separation and, sequentially, the analysis of polymorphisms and genetic diversity within the population under study. Native and reducing conditions were applied and the results are shown in Figure 2.

The unreduced electrophoretic profile (Figure 2a) showed high molecular weight proteins with a molecular size greater than 148 kDa (most likely hexamers and trimers of globulins), an intermediate set of proteins between 36 to 50 kDa and several less intense bands under 36 kDa. Comparing the native and the reduced profiles (Figure 2a,b), it is possible to verify that the bands corresponding to the high molecular weight proteins (>148 kDa) became very faint, as well as the protein bands having a molecular weight equal to or slightly greater than 50 kDa. On the other hand, well-marked protein bands appeared between 22 and 36 kDa, between 16 and 22 kDa, and between 6 and 16 kDa (Figure 2b). First, it should be noted that molecular weights of proteins are not estimated with high precision when determined by their relative mobility in SDS-PAGE [22]. Nevertheless, this behavior can be partly explained by the 11S legumin-type globulins in hazelnut. In fact, these are proteins with a theoretical molecular weight of 59 kDa that form functional hexameric structures, interacting non-covalently and arranged in an open ring conformation with 360 kDa. Each subunit is constituted by an acidic polypeptide (30–40 kDa) linked to a basic polypeptide (~20 kDa) by a disulfide bond [23]. At least, the split of the major band near to 50 kDa into two major bands between 22 and 36 kDa (acid subunit) and between 16 and 22 kDa (basic subunit) was already reported in the literature [24]. In addition, despite the legumins (hexamers) and vicilins (trimers with small percentage of hexamers) display similar conformational structures, the primary structure of vicilins does not comprise cysteine residues [25].

Within the studied varieties, we verified differences in the electrophoretic profiles. For example, the varieties ‘Fertile de Coutard’, ‘Grossal’ and ‘Longue d’Espagne’ stand out for the difference in intensity and/or presence/absence of the above-mentioned major proteins. In addition, differences among varieties are most noticeable in the native profile and particularly for an apparent molecular weight below 36 kDa. From the native electrophoretic profile, up to 35 representative bands were found, of which 29 were polymorphic and 6 were monomorphic. For the purposes of genetic analysis, i.e., to check if the studied hazelnut varieties represent a genetically diverse group and further identify any variety-dependent allergenic potential for susceptible individuals, the electrophoretic profiles were divided into three molecular weight ranges, as previously described [26]. Eight bands were detected in zone A, above 50 kDa, seven bands were detected in zone B between 36 and 50 kDa, and twenty bands in zone C, bellow 36 kDa. The bands were named with the capital letter referring to the gel zone followed by a number indicating the relative mobility in SDS-PAGE: A1 is the first band of zone A, B1 is the first band of zone B, and so on (Table S2, Supplementary Material). For zone A, the band A4 was the most frequent, being present in 76.9% of the varieties, while the remaining bands showed a prevalence ranging from 15.4 to 46.2%. In zone B, there were three monomorphic bands, B9, B14 and B15. In zone C, four rare bands were detected, which were only present in the ‘Longue d’Espagne’ (C16 and C21), Segorbe (C23) and ‘Fertile de Coutard’ (C28) varieties, so with a frequency of 7.7%. Furthermore, three bands, C19, C22 and C24, were monomorphic, and the frequencies of the remaining bands ranged between 7.7 and 84.6% (Table S3, Supplementary Material). The hazelnut collection evaluated was highly polymorphic for its storage proteins as the percentage of polymorphic loci was 82.9%, showing also considerable diversity (h = 0.291 ± 0.029). In fact, using other molecular markers, several studies have consistently shown a great genetic diversity for this tree nut [9,27].

Cluster analyses based on the presence or absence of protein alleles, using the unweighted pairwise group method with arithmetical average (UPGMA), were performed with different algorithms. The results were used to generate dendrograms displaying the hierarchical associations among all the studied varieties. Similar results were generated with the different tree-building algorithms. One dendrogram based on Jaccard’s similarity coefficient is shown in Figure 2c. The cophenetic correlation coefficient for this matrix was the highest (r = 0.846) compared to the other analyzed coefficients, which indicates the clustering fitted the data better [28]. The dendrogram grouped the varieties into different clusters. ‘Negreta’ and ‘Tonda di Giffoni’ were the most similar pairs (0.944) among all the varieties studied (Table S4, supplementary material). By contrast, ‘Grada de Viseu’ and ‘Longue d’Espagne’ (0.269) were the least similar. It was not possible to identify any clear relationship between the genetic similarity and the geographical origin of the varieties. The hazelnut varieties were grown under similar soil and climate conditions and proved to be a genetically diverse group, thus suitable for this study.

### 3.3. Allergenic Potential

**Figure 3:**
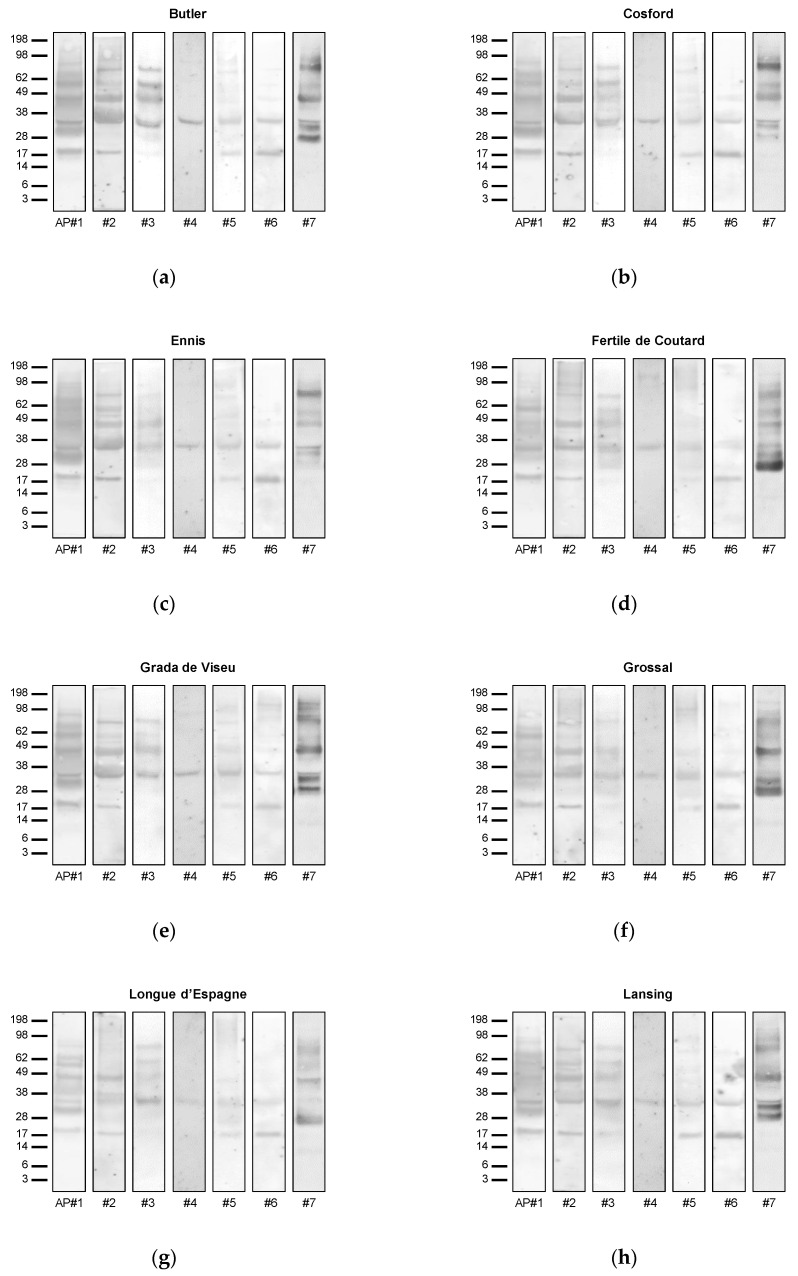

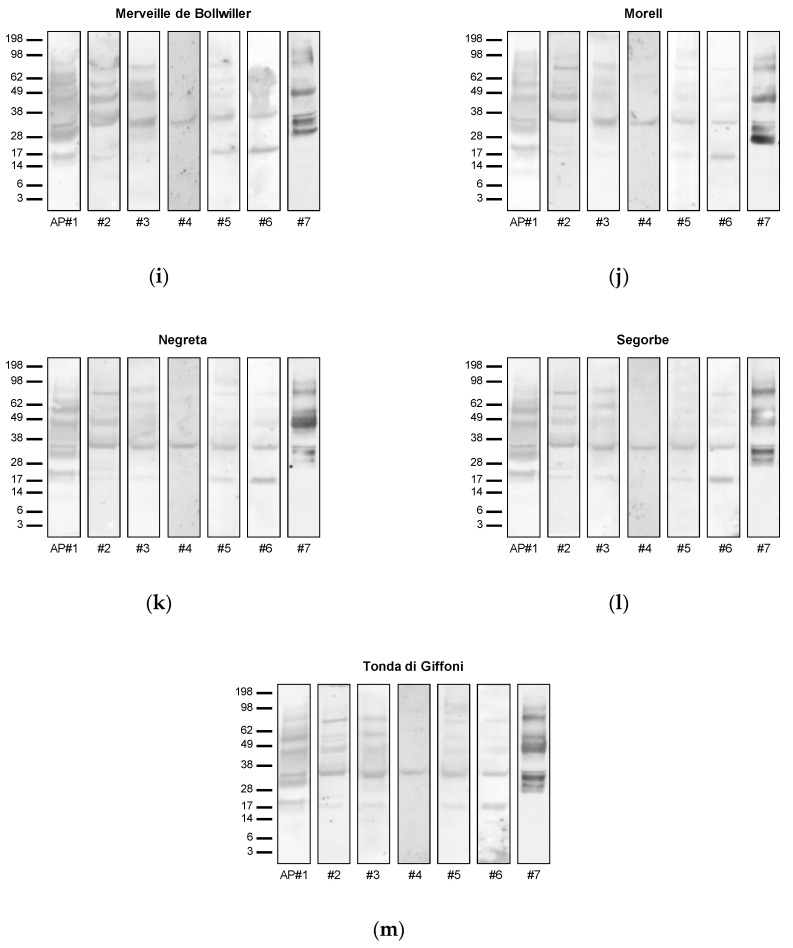
(**a**–**m**) Immunoblot of protein extracts from hazelnut varieties using individual sera from allergic patients. The secondary antibody was HRP-conjugated monoclonal anti-human IgE. Sizes (in kDa) of protein molecular weight markers (SeeBlue™ Plus2 Pre-stained Protein Standard, Thermo Fisher Scientific, Waltham, MA, USA) are shown on the left.

In order to test the allergenic potential of the different hazelnut varieties, the sera of seven allergic patients were used (Figure 3).

All hazelnut varieties showed similar IgE-reactivity profiles for each of the analyzed serum. However, differences can be observed when the IgE-binding profiles of different patients are compared. This is the case of the serum from allergic patient #4, which only presented immunoreactivity with one protein at approximately 34 kDa, being this protein recognized by the sera of all tested patients in all hazelnut varieties (Figure 3). Under the reducing conditions used, this band seems to correspond to the acidic subunit of legumin (Cor a 9), being in good agreement with data reported in other studies [23]. Based on the literature and considering the data described herein, Cor a 9 (acidic subunit) seems to correspond to the major hazelnut allergen for several individuals, as previously reported elsewhere [23]. With a molecular weight close to 17 kDa, a band was also detected in 4 out of the 7 analyzed sera, namely for individuals #1, #2, #5 and #6, which resembles to the hazelnut allergen Cor a 1.04 [29]. In this study, this protein is recognized by more than 50% of the tested sera of hazelnut-allergic patients, thus confirming its classification as a major allergen in hazelnut. The immunoreactive profile of the different hazelnut varieties for the different sera also showed other important allergens, namely at a molecular weight slightly higher than 62 kDa (allergic patients #1, #2, #3, faint in #5/#6 and #7), and approximately at 62 kDa (allergic patients #1, #2, #3 and #7), 49 kDa (allergic patients #1, #2, #3, #6 and #7) and 28 kDa (allergic patients #1 and #7), which is in accordance with previous studies aiming at identifying common allergenic structures in hazelnut [30,31]. A few faint immunoreactive bands with molecular weights between 62 and 98 kDa were also observed in the sera of some allergic patients, namely #1 and #7, which were posteriorly tested by the 2-DE-based proteomic approach in the present work.

Curiously, despite patient #3 presenting the same immunoreactivity profile among distinct hazelnut varieties, the intensity of some proteins appears to be quite different. For example, the immunorecognition of proteins of 34, 49, 62 and ~80 kDa was much more intense for ‘Buttler’ and ‘Cosford’, comparing to the other varieties (Figure 3), which could implicate a stronger immune response upon the consumption of these specific hazelnut varieties. Patient #7 also evidenced strong IgE-reactivity to different proteins, depending on the variety tested (Figure 3). This serum was highly reactive to a protein at ~80 kDa in ‘Cosford’, while in ‘Fertile de Coutard’, it reacted the most with a protein at ~28 kDa. Serum from patient #7 was less immunoreactive to proteins from ‘Ennis’ and ‘Merveille de Bollwiller’ varieties. However, it is important to highlight that this evaluation is based on qualitative information provided by the immunoblot, and as such, it is always susceptible to visual interpretation. Moreover, this type of assay is highly dependent on the type of sera used (age, sex, presence of concomitant diseases, genetic heritage, among others), which can be prone to bias. Even considering the same patient, the serum can present differences depending on several intrinsic and environmental factors [32]. To support this evaluation, 2-DE-based proteomic approach was used in order to provide unequivocal identification of several proteins [21].

**Figure 4:**
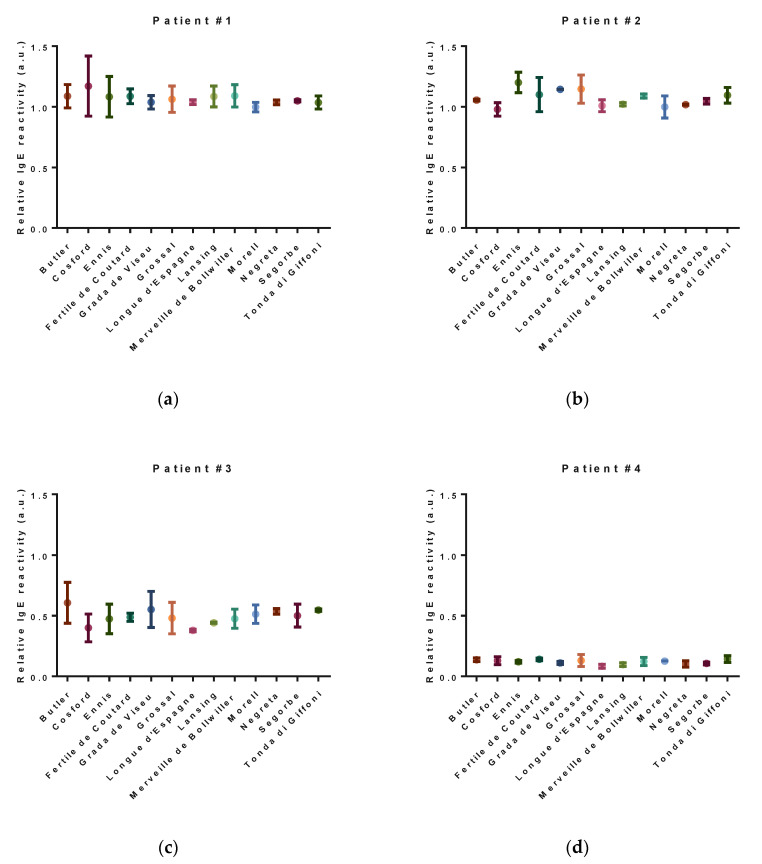

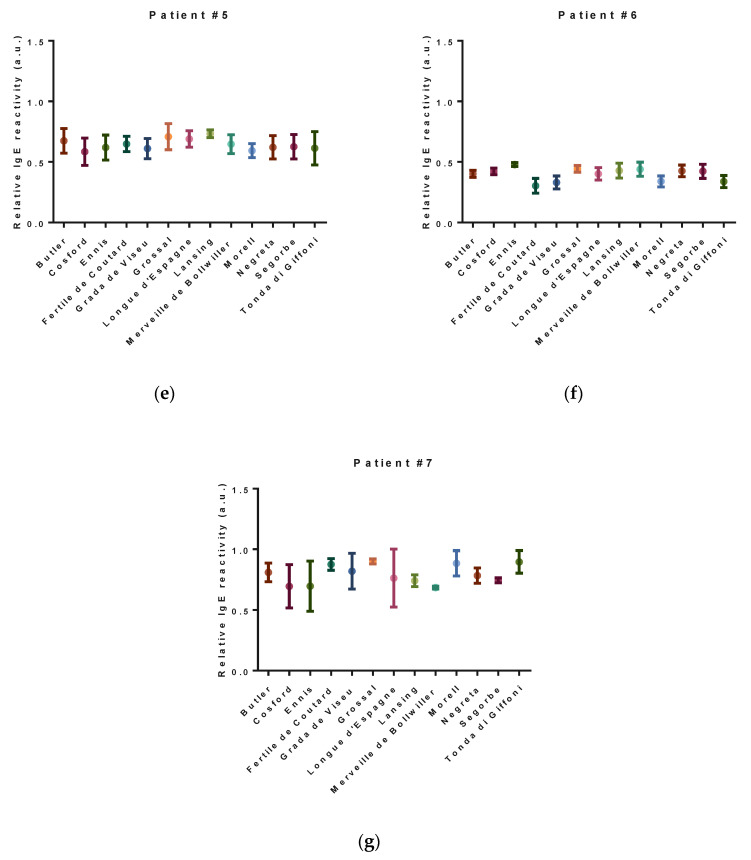
(**a**–**g**) Relative IgE-reactivity of the different hazelnut varieties under study using the sera of seven allergic individuals. No significant differences were detected (*p* < 0.05).

The relative IgE-reactivity for each variety is shown in Figure 4.

There are no significant differences among the different hazelnut varieties in terms of relative IgE-reactivity measured using the sera from seven allergic patients, although punctual differences among sera’ immune responses to specific proteins could be noticed. On the other hand, the immunoreactivity varied significantly within the allergic population under study, presenting for patient #4 and patients #1 and #2, the lowest and the highest values, respectively. Our analysis focused on the total IgE-reactivity of each variety (relative to loading control) suggests a similar allergenic potential among the different hazelnut varieties analyzed. Nevertheless, some differences may exist that may not be accounted for, due to the limitation of the immunoblot technique. In particular, those related to the possibility of variations in the primary structure of proteins of the different varieties result in secondary conformations that alter the IgE-binding properties [33,34] and, in this sense, complementary assays, such as the basophil activation test (BAT), can be used in future studies to provide another type of data on the subject [35,36].

Comparable results were obtained by Vieths, et al. [37], who evaluated the allergenic activity of six hazelnut varieties using a serum pool from four hazelnut allergic patients by the enzyme allergosorbent test (EAST). The authors also reported no differences in the IgE-binding capacity of different hazelnut varieties. Later, EAST inhibition experiments, comparing the IgE-binding potency of five hazelnut varieties using a pooled serum of 13 patients’ sera of known allergen specificity, also indicated minor differences among varieties [38].

### 3.4. Allergen Identification

**Figure 5:**
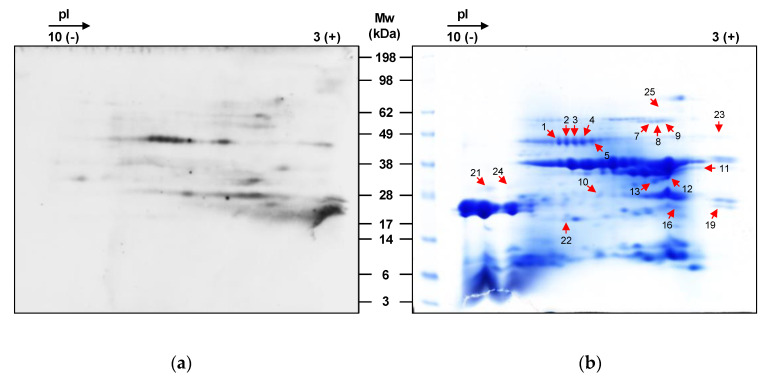
(**a**) 2D immunoblot of ‘Merveille de Bollwiller’ hazelnut variety with a serum pool of the seven allergic patients enrolled in the study. (**b**) On the right, it is shown the ‘Merveille de Bollwiller’ 2DE profile and the immunoreactive spots analyzed by nano LC-MS/MS-based peptide mass fingerprinting approach.

The identification of allergenic proteins of hazelnut was performed by 2-DE-based immunoblotting using a serum pool of the seven allergic patients enrolled in this study (Figure 5a).

The variety ‘Merveille de Bollwiller’ was selected for this analysis because it previously showed the lowest Cq values targeting the Cor a 8, Cor a 9, and Cor a 14 genes. The immunoreactive spots were excised from the 2-DE gels and identified by nano LC-MS/MS-based peptide mass fingerprinting after in-gel tryptic digestion. Table 2 shows the names, the assigned molecular function, the species in which they were described, the accession numbers, the molecular weights, the Mascot scores, the number of significant unique sequences identified and the sequence coverage of the proteins identified in ‘Merveille de Bollwiller’. Hazelnut proteins were clearly separated with most forming a single spot on the 2-DE gel (Figure 5b). The presence of multiple components with close molecular weights and isoelectric points (pI) was also observed, highlighting the complexity of the hazelnut proteome. Actually, the existence of several proteoforms, including many glycoforms, and post-translationally protease-cleaved subunits contributes to this heterogeneity [21,39].

Most of the identified proteins relate to the biological functions of nutrient reservoir, whereas a defense response-related protein was identified in just one spot. The Cor a 9 allergen was identified in the spots 7, 8, 9, 10, 11, 12, 13, 16, 19 and 25, having been the most frequently identified protein. Cor a 9, also known as corylin, is a 11S legumin-type globulin, being also the major component of the protein fraction of hazelnut. Functional Cor a 9 is constituted by an acidic polypeptide chain linked to a basic polypeptide chain by a disulfide bond. The identified allergen is the acidic subunit, after the cleavage and the reduction of the main protein [23]. In this work, the results seem to corroborate this hypothesis [23] since the proteins identified as Cor a 9 allergen are all located in the acidic zone of the 2-DE gel. However, these findings contrast with another work reporting that only the basic subunit exhibited IgE-affinity [5]. A 48-kDa glycoprotein, commonly known as Cor a 11 (7S vicilin-type globulin), was identified in 8 out of 19 spots. Mature 7S globulins are typically trimeric proteins, but they can also occur as hexamers, with molecular weights that can range from 150 to 190 kDa, having subunits with molecular weights within 40–80 kDa each [40,41]. As for Cor a 9, Cor a 11 was identified in several spots displaying a different molecular weight. Moreover, the spots 1, 2, 3, 4 and 5 have an identical apparent molecular weight, but show a different isoelectric point, which is probably related to post-translational modifications (PTM), namely glycosylation. This is the characteristic PTM of vicilins, thus confirming the identity of Cor a 11 [42]. The major allergen variant Cor a 1.0403 was identified in spot 22. Cor a 1.04 is the only isoallergen from Cor a 1 that is present in the hazelnut seed, while Cor a 1.01 to Cor a 1.03 appear in the hazel pollen (Roux et al., 2003). Their designation as isoallergens results from their similar molecular weights (~17 kDa), identical biological function, and great amino acid sequence identity (≥67%) [43].

Through the bottom-up strategy based on in-gel trypsin digestion and mass spectrometry analysis, it was possible to identify three major hazelnut allergens, namely Cor a 1.04, Cor a 9 and Cor a 11. Cor a 9 was identified in 10 out of 19 spots. In the light of the results discussed above, the entire population of allergic patients enrolled in this study recognized Cor a 9 allergen, while only 60% recognized Cor a 1.04, which is in good agreement with their classification as major allergens in hazelnut. Curiously, no peptides/protein of Cor a 8 were identified by LC-MS/MS or by immunoblot. Cor a 8 is a nsLTP protein with high geographical importance at the Mediterranean area, so it was expected that some of the patients’ sera from this region would be sensitized to Cor a 8. However, this lack of sensitization might be explained by the high sensitization to birch pollen (Bet v 1) homologue protein in hazelnut (Cor a 1.04), suggesting some protective effects against LTP-allergic symptoms in these patients [44].

## 4. Conclusions

Hazelnut is of global agricultural and economic significance, being one of the most commonly consumed nuts. Currently, special attention is given to food-induced allergies, in which hazelnut allergy is highlighted. Due to hazelnut genetic diversity with the existence of many varieties, variety-dependent differences in the IgE-binding properties may be suspected. Nevertheless, our results do not support this hypothesis; although some minor differences were found at the level of genes encoding important allergens, namely Cor a 8, Cor a 9 and Cor a 14 for the 13 hazelnut varieties under study, these results were not reflected at the level of IgE-reactivity analysis using sera from allergic individuals. Cor a 1.04 and Cor a 9 were the predominant immunoreactive allergens within the tested sera, while no IgE-binding was observed for Cor a 8. From this study, none of the hazelnut varieties seems to be promising in terms of potential hypoallergenicity. Therefore, it seems that the administration of small amounts of hazelnut to induce desensitization using putative hypoallergenic hazelnut varieties is not practicable and the conventional eviction dietary treatment will hold its ground for the time being.

## Figures and Tables

**Table 1:** Data from hazelnut allergic patients.

Patient	Sex	Age	Hazelnut Allergy		Concomitant Other Food Allergies	Concomitant Allergy to Aeroallergens
			Symptoms	CAP specific IgE (f17; kUA/L)		
1	M	4	Urticaria	75.9	Yes	Yes
2	F	18	Urticaria, anaphylaxis	35.3	Yes	Yes
3	M	71	Eczema, rhinitis, asthma	40.9	Yes	Yes
4	M	19	Angioedema	55.1	Yes	Yes
5	F	28	Angioedema, anaphylaxis	21.6	Yes	Yes
6	F	45	Rhinitis, angioedema	65.4	Yes	Yes
7	M	29	Rhinitis	21.3	Yes	No

**Table 2:** Mass spectrometry-identified proteins of the ‘Merveille de Bollwiller’ (*Corylus avellana* L.) 2DE profile, corresponding to immunoreactive spots.

Spot *	Protein	Function	UniProt ^†^	Mw (Da)	Score	Significant Unique Sequences	MS Coverage (%)
1	48-kDa glycoprotein	Nutrient reservoir	Q8S4P9	50,856	1362	17	39
2	48-kDa glycoprotein	Nutrient reservoir	Q8S4P9	50,856	1351	18	45
3	48-kDa glycoprotein	Nutrient reservoir	Q8S4P9	50,856	1377	19	48
4	48-kDa glycoprotein	Nutrient reservoir	Q8S4P9	50,856	1104	19	47
5	48-kDa glycoprotein	Nutrient reservoir	Q8S4P9	50,856	1353	19	47
7	Cor a 9 allergen	Nutrient reservoir	A0A0A0P7E3	58,837	789	11	26
8	Cor a 9 allergen	Nutrient reservoir	A0A0A0P7E3	58,837	463	10	25
9	Cor a 9 allergen	Nutrient reservoir	A0A0A0P7E3	58,837	389	7	30
10	Cor a 9 allergen	Nutrient reservoir	A0A0A0P7E3	58,837	708	14	35
11	Cor a 9 allergen	Nutrient reservoir	A0A0A0P7E3	58,837	186	4	18
12	Cor a 9 allergen	Nutrient reservoir	A0A0A0P7E3	58,837	616	9	29
13	Cor a 9 allergen	Nutrient reservoir	A0A0A0P7E3	58,837	377	7	19
16	Cor a 9 allergen	Nutrient reservoir	A0A0A0P7E3	58,837	676	9	27
19	Cor a 9 allergen	Nutrient reservoir	A0A0A0P7E3	58,837	669	10	30
21	48-kDa glycoprotein	Nutrient reservoir	Q8S4P9	50,856	299	7	16
22	Variant Cor a 1.0403	Defense response	Q9FPK3	17,527	406	11	76
23	48-kDa glycoprotein	Nutrient reservoir	Q8S4P9	50,856	735	13	31
24	48-kDa glycoprotein	Nutrient reservoir	Q8S4P9	50,856	140	5	16
25	Cor a 9 allergen	Nutrient reservoir	A0A0A0P7E3	58,837	375	4	21

* Spots are shown in Figure 5b. ^†^ https://www.uniprot.org/. Mw, molecular weight; MS coverage, percentage of amino acids in sequence matched by peptides detected by mass spectrometry.

## Supplementary Materials

The following are available online at https://www.mdpi.com/2072-6643/12/7/2100/s1, Figure S1: Example of (**a**) 1D and (**b**) 2D immunoblots using pooled sera from non-allergic individuals as negative controls. No IgE reactivity was observed; Figure S2: Sequencing alignments of Cor a 8 gene for all tested hazelnut varieties cultivars. Primers Cor 8F/Cor 8R delimit the DNA region amplified by real-time PCR assays; Figure S3: Sequencing alignments of Cor a 9 gene for all tested hazelnut varieties cultivars. Primers Cor 9F/Cor 9R delimit the DNA region amplified by real-time PCR assays; Figure S4: Sequencing alignments of Cor a 14 gene for all tested hazelnut varieties cultivars. Primers Cor 14F/Cor 14R delimit the DNA region amplified by real-time PCR assays; Table S1: Primers targeting *Corylus avellana* L. lipid transfer protein precursor mRNA, *Corylus avellana* L. 11S globulin-like protein mRNA and *Corylus avellana* L. 2S albumin mRNA, encoding Cor a 8, Cor a 9 and Cor a 14, respectively; Table S2: Reference mobility of each band detected for band pattern matching using CLIQS 1D Pro software (TotalLab, UK); Table S3: Frequencies of each band detected in hazelnut electrophoretic profile; Table S4: Similarity matrix of the studied hazelnut varieties based on storage proteins polymorphism.

## References

[1] Yu W., Freeland D.M.H., Nadeau K.C. (2016). Food allergy: Immune mechanisms, diagnosis and immunotherapy. Nat. Rev. Immunol..

[2] Benedé S., Blazquez A.B., Chiang D., Tordesillas L., Berin M.C. (2016). The rise of food allergy: Environmental factors and emerging treatments. EBioMedicine.

[3] Dhondalay G.K., Rael E., Acharya S., Zhang W., Sampath V., Galli S.J., Tibshirani R., Boyd S.D., Maecker H., Nadeau K.C. (2018). Food allergy and omics. J. Allergy Clin. Immunol..

[4] Costa J., Mafra I., Carrapatoso I., Oliveira M.B.P.P. (2015). Hazelnut allergens: Molecular characterisation, detection and clinical relevance. Crit. Rev. Food Sci. Nutr..

[5] Nitride C., Mamone G., Picariello G., Mills C., Nocerino R., Canani R.B., Ferranti P. (2013). Proteomic and immunological characterization of a new food allergen from hazelnut (Corylus avellana). J. Proteom..

[6] Burney P., Summers C., Chinn S., Hooper R.L., Van Ree R., Lidholm J. (2010). Prevalence and distribution of sensitization to foods in the European community respiratory health survey: A europrevall analysis. Allergy.

[7] Pfeifer S., Bublin M., Dubiela P., Hummel K., Wortmann J., Hofer G., Keller W., Radauer C., Sommergruber K.H. (2015). Cor a 14, the allergenic 2S albumin from hazelnut, is highly thermostable and resistant to gastrointestinal digestion. Mol. Nutr. Food Res..

[8] Mills E.N.C., Jenkins J., Marigheto N., Belton P.S., Gunning A.P., Morris V.J. (2002). Allergens of the cupin superfamily. Biochem. Soc. Trans..

[9] Öztürk S.C., Balık S.K., Kızılcı G., Duyar Ö., Doğanlar S., Frary A., Balık H.I. (2017). Molecular genetic diversity of the Turkish national hazelnut collection and selection of a core set. Tree Genet. Genomes.

[10] Garino C., Locatelli M., Coisson J.D., D’Andrea M., Cereti E., Travaglia F., Arlorio M. (2013). Gene transcription analysis of hazelnut (Corylus avellanaL.) allergens Cor a 1, Cor a 8 and Cor a 11: A comparative study. Int. J. Food Sci. Technol..

[11] Korte R., Happe J., Brümmer I., Brockmeyer J. (2017). Structural Characterization of the Allergenic 2S Albumin Cor a 14: Comparing Proteoform Patterns across Hazelnut Cultivars. J. Proteome Res..

[12] Pastorello E.A., Vieths S., Pravettoni V., Farioli L., Trambaioli C., Fortunato D., Lüttkopf D., Calamari M., Ansaloni R., Scibilia J. (2002). Identification of hazelnut major allergens in sensitive patients with positive double-blind, placebo-controlled food challenge results. J. Allergy Clin. Immunol..

[13] Ribeiro M., Nunes F.M., Guedes S.D.M., Domingues P., Silva A.M., Carrillo J.M., Rodríguez-Quijano M., Branlard G., Igrejas G. (2015). Efficient chemo-enzymatic gluten detoxification: Reducing toxic epitopes for celiac patients improving functional properties. Sci. Rep..

[14] Portugal C., Pinto L., Ribeiro M., Tenorio C., Igrejas G., Ruiz-Larrea M.F. (2015). Potential spoilage yeasts in winery environments: Characterization and proteomic analysis of Trigonopsis cantarellii. Int. J. Food Microbiol..

[15] Ribeiro M., Seabra L., Ramos A., Santos S., Pinto-Carnide O., Carvalho C., Igrejas G. (2012). Polymorphism of the storage proteins in Portuguese rye (Secale cereale L.) populations. Hereditas.

[16] Ribeiro M., Bancel E., Faye A., Dardevet M., Ravel C., Branlard G., Igrejas G. (2013). Proteogenomic characterization of novel x-type high molecular weight glutenin subunit 1Ax1.1. Int. J. Mol. Sci..

[17] Romero-Calvo I., Ocón B., Martínez-Moya P., Suárez M.D., Zarzuelo A., Martínez-Augustin O., De Medina F.S. (2010). Reversible Ponceau staining as a loading control alternative to actin in Western blots. Anal. Biochem..

[18] Peakall R., Smouse P.E. (2012). GenAlEx 6.5: Genetic analysis in Excel. Population genetic software for teaching and research--an update. Bioinformatics.

[19] Rolph F.J. (2000). NTSYS, numerical taxonomy and multivarietal analysis system (2.1): User guide. Biostatistics.

[20] Marzano V., Tilocca B., Fiocchi A.G., Vernocchi P., Mortera S.L., Urbani A., Roncada P., Putignani L. (2020). Perusal of food allergens analysis by mass spectrometry-based proteomics. J. Proteom..

[21] Mouzo D., Bernal J., López-Pedrouso M., Franco D., Zapata C. (2018). Advances in the Biology of Seed and Vegetative Storage Proteins Based on Two-Dimensional Electrophoresis Coupled to Mass Spectrometry. Molecules.

[22] Gao L., Ma W., Chen J., Wang K., Li J., Wang S., Békés F., Appels R., Yan Y. (2010). Characterization and comparative analysis of wheat high molecular weight glutenin subunits by SDS-PAGE, RP-HPLC, HPCE, and MALDI-TOF-MS. J. Agric. Food Chem..

[23] Beyer K., Grishina G., Bardina L., Grishin A., Sampson H.A. (2002). Identification of an 11S globulin as a major hazelnut food allergen in hazelnut-induced systemic reactions. J. Allergy Clin. Immunol..

[24] Guo F., Kothary M.H., Wang Y., Yu X., Howard A.J., Fu T.J., Zhang Y. (2008). Purification and crystallization of Cor a 9, a major hazelnut allergen. Acta Crystallogr. Sect. F Struct. Boil. Cryst. Commun..

[25] Rougé P., Brunet E., Borges J.-P., Jauneau A., Saggio B., Bourrier T., Rancé F., Didier A., Barre A. (2011). Les protéines à motif cupine: Allergènes majeurs des graines. Revue Française d’Allergologie.

[26] Singh N.P., Matta N.K. (2015). Phylogenetic relationship and germplasm evaluation of different taxa of the genusCucurbitausing seed storage protein profiling. Plant. Biosyst. Int. J. Deal. all Asp. Plant. Biol..

[27] Boccacci P., Akkak A., Botta R. (2006). DNA typing and genetic relations among European hazelnut (Corylus avellana L.) cultivars using microsatellite markers. Genome.

[28] Ribeiro M., Freitas M., Domínguez-Perles R., Barros A.I.R.N.A., Ferreira-Cardoso J., Igrejas G. (2020). Nutriproteomics survey of sweet chestnut (Castanea sativa Miller) genetic resources in Portugal. Food Bioscience.

[29] Roux K.H., Teuber S.S., Sathe S.K. (2003). Tree nut allergens. Int. Arch. Allergy Immunol..

[30] Dooper M., Plassen C., Holden L., Moen L.H., Namork E., Egaas E. (2008). Antibody binding to hazelnut (Corylus avellana) proteins: The effects of extraction procedure and hazelnut source. Food Agric. Immunol..

[31] Platteau C.M.F., Bridts C.H., Daeseleire E.A., De Loose M.R., Ebo D., Taverniers I. (2010). Comparison and Functional Evaluation of the Allergenicity of Different Hazelnut (Corylus avellana) Protein Extracts. Food Anal. Methods.

[32] Stemeseder T., Klinglmayr E., Moser S., Lang R., Himly M., Oostingh G.J., Zumbach J., Bathke A.C., Hawranek T., Gadermaier G. (2017). Influence of intrinsic and lifestyle factors on the development of IgE sensitization. Int. Arch. Allergy Immunol..

[33] Barre A., Sordet C., Culerrier R., Rancé F., Didier A., Rougé P. (2008). Vicilin allergens of peanut and tree nuts (walnut, hazelnut and cashew nut) share structurally related IgE-binding epitopes. Mol. Immunol..

[34] Robotham J.M., Hoffman G.G., Teuber S.S., Beyer K., Sampson H.A., Sathe S.K., Roux K.H. (2009). Linear IgE-epitope mapping and comparative structural homology modeling of hazelnut and English walnut 11S globulins. Mol. Immunol..

[35] Hemmings O., Kwok M., McKendry R., Santos A.F. (2018). Basophil activation test: Old and new applications in allergy. Curr. Allergy Asthma Rep..

[36] Volpicella M., Leoni C., Dileo M.C., Ceci L.R. (2019). Progress in the analysis of food allergens through molecular biology approaches. Cells.

[37] Vieths S., Hoffmann A., Holzhauser T., Müller U., Reindl J., Haustein D. (1998). Factors influencing the quality of food extracts forin vitroandin viiodiagnosis. Allergy.

[38] Wigotzki M., Steinhart H., Paschke A. (2000). Influence of varieties, storage and heat treatment on ige-binding proteins in hazelnuts (Corylus avellana). Food Agric. Immunol..

[39] Nitride C., Picariello G., Mamone G., Ferranti P., Colgrave M.L. (2017). Proteomics of Hazelnut (Corylus avellana). Proteomics in Food Science.

[40] Breiteneder H., Radauer C. (2004). A classification of plant food allergens. J. Allergy Clin. Immunol..

[41] Alessandri S., Sancho A., Vieths S., Mills C.E.N., Wal J.-M., Shewry P.R., Rigby N., Hoffmann-Sommergruber K. (2012). High-throughput NMR assessment of the tertiary structure of food allergens. PLoS ONE.

[42] Graham D.R.M., Mitsak M.J., Elliott S.T., Chen D., Whelan S.A., Hart G.W., Van Eyk J.E., Whelan S.A. (2008). Two-dimensional gel-based approaches for the assessment of N-Linked and O-GlcNAc glycosylation in human and simian immunodeficiency viruses. Proteomics.

[43] Chapman M.D., Pomés A., Breiteneder H., Ferreira F. (2007). Nomenclature and structural biology of allergens. J. Allergy Clin. Immunol..

[44] Asero R., Piantanida M., Pinter E., Pravettoni V. (2017). The clinical relevance of lipid transfer protein. Clin. Exp. Allergy.

