# Supplementary material for: Natural Variation of Hazelnut Allergenicity: Is There Any Potential for Selecting Hypoallergenic Varieties?
Source: Nutrients. 2020 Jul 16;12(7):2100. doi: 10.3390/nu12072100 (PMC7400875; doi:10.3390/nu12072100)
Supplement: Supplementary file 1 [file nutrients-12-02100-s001.pdf]

Supplementary Material

Article

Natural variation of hazelnut allergenicity: is there any potential for selecting hypoallergenic varieties?

Miguel Ribeiro [1,2,3], Joana Costa [4], Isabel Mafra [4], Sandra Cabo [5], Ana Paula Silva [6], Berta Gonçalves [5], Mélanie Hillion [7,8], Michel Hébraud [7,8], Gilberto Igrejas [1,2,3,*]

[1] Department of Genetics and Biotechnology, University of Trás-os-Montes and Alto Douro, 5000-801 Vila Real, Portugal; jmribeiro@utad.pt
[2] Functional Genomics and Proteomics Unity, University of Trás-os-Montes and Alto Douro, 5000-801 Vila Real, Portugal
[3] LAQV-REQUIMTE, Faculty of Science and Technology, University Nova of Lisbon, Lisbon, Caparica, Portugal
[4] REQUIMTE-LAQV, Faculdade de Farmácia, Universidade do Porto, Rua de Jorge Viterbo Ferreira, 228, 4050-313 Porto, Portugal; jbcosta@ff.up.pt, isabel.mafra@ff.up.pt
[5] Department of Biology and Environment, Centre for the Research and Technology of Agro-Environmental and Biological Sciences (CITAB), University of Trás-os-Montes and Alto Douro, 5000-801, Vila Real, Portugal; scsc90@hotmail.com, bertag@utad.pt
[6] Department of Agronomy, Centre for the Research and Technology of Agro-Environmental and Biological Sciences (CITAB), University of Trás-os-Montes and Alto Douro, Vila Real, Portugal; asilva@utad.pt
[7] Université Clermont Auvergne (UCA), Institut National de Recherche pour l'Agriculture, l'Alimentation et l'Environnement (INRAE), Unité Mixte de Recherche (UMR) Microbiologie Environnement Digestif Santé (MEDiS); melanie.hillion@inrae.fr, michel.hebraud@inrae.fr
[8] Metabolomic and Proteomic Exploration Facility (PFEM)2, F-63122 Saint-Genès Champanelle, France
* Correspondence: gigrejas@utad.pt; Tel.: 00351 259350530

Index

Table S1. Primers targeting *Corylus avellana* L. lipid transfer protein precursor mRNA, *Corylus avellana* 11S globulin-like protein mRNA and *Corylus avellana* 2S albumin mRNA, encoding Cor a 8, Cor a 9 and Cor a 14, respectively. 3

Fig. S1. Example of (**a**) 1D and (**b**) 2D immunoblots using pooled sera from non-allergic individuals as negative controls. No IgE reactivity was observed. 4

Fig. S2. Sequencing alignments of Cor a 8 gene for all tested hazelnut varieties cultivars. Primers Cor 8F/Cor 8R delimit the DNA region amplified by real-time PCR assays. 5

Fig. S3. Sequencing alignments of Cor a 9 gene for all tested hazelnut varieties cultivars. Primers Cor 9F/Cor 9R delimit the DNA region amplified by real-time PCR assays. 6

Fig. S4. Sequencing alignments of Cor a 14 gene for all tested hazelnut varieties cultivars. Primers Cor 14F/Cor 14R delimit the DNA region amplified by real-time PCR assays. 7

Table S2. Reference mobility of each band detected for band pattern matching using CLIQS 1D Pro software (TotalLab, UK). 8

Table S3. Frequencies of each band detected in hazelnut electrophoretic profile. 10

Table S4. Similarity matrix of the studied hazelnut varieties based on storage proteins polymorphism. 11

Table S1. Primers targeting *Corylus avellana* L. lipid transfer protein precursor mRNA, *Corylus avellana* 11S globulin-like protein mRNA and *Corylus avellana* 2S albumin mRNA, encoding Cor a 8, Cor a 9 and Cor a 14, respectively.

Coding protein	Primers	Sequence (5′-3′)	Amplicon (bp)	NCBI accession number
Cor a 8	Real-time PCR			
	Cor 8F	AAGTTGGTATGCGCGGTCCTCT	118	AF329829.1
	Cor 8R	CGTTCTTCAGGTAGAGCACGCA		
	Sequencing			
	Cor 8FS	TGGGTAGCCTTAAGTTGGTATGC	342	
	Cor 8RS	TCACGTTGTTGCAGTTGGTGGAG		
Cor a 9	Real-time PCR			
	Cor 9F	CAGGAGACCACCTTGGTTCGC	169	AF449424.1
	Cor 9R	GCATAGTGATGGCCCTAAACCCT		
	Sequencing			
	Cor 9FS	TCCTCTTGCCGGACGAACCTCA	339	
	Cor 9RS	TGTAGTAAAGGTTGCCCTTGTGC		
Cor a 14	Real-time PCR			
	Cor 14F	AGGGTCACCGCTAGATATAAATGG	122	FJ358504.1
	Cor 14R	ACAGCCTACAAAGCCTCGAACTA		
	Sequencing			
	Cor 14FS	ACATGAGGCAGCAAAGCCAGTA	417	
	Cor 14RS	TCAAGAGACAGCCTACAAAGCCT		

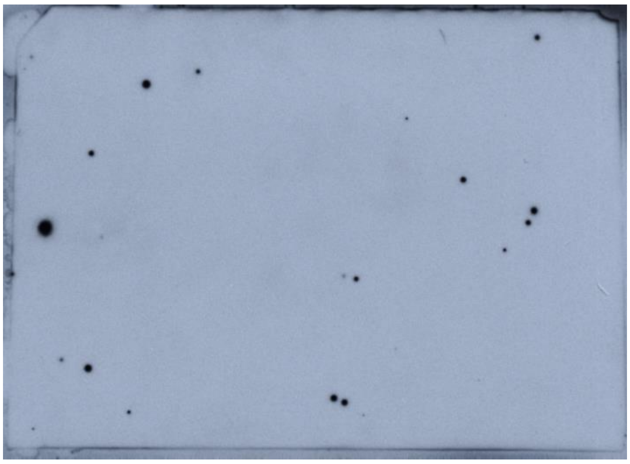

(**a**)

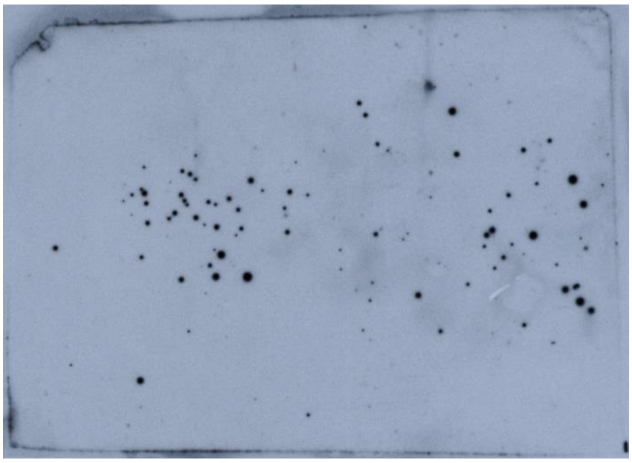

(**b**)

Fig. S1. Example of (**a**) 1D and (**b**) 2D immunoblots using pooled sera from non-allergic individuals as negative controls. No IgE reactivity was observed.

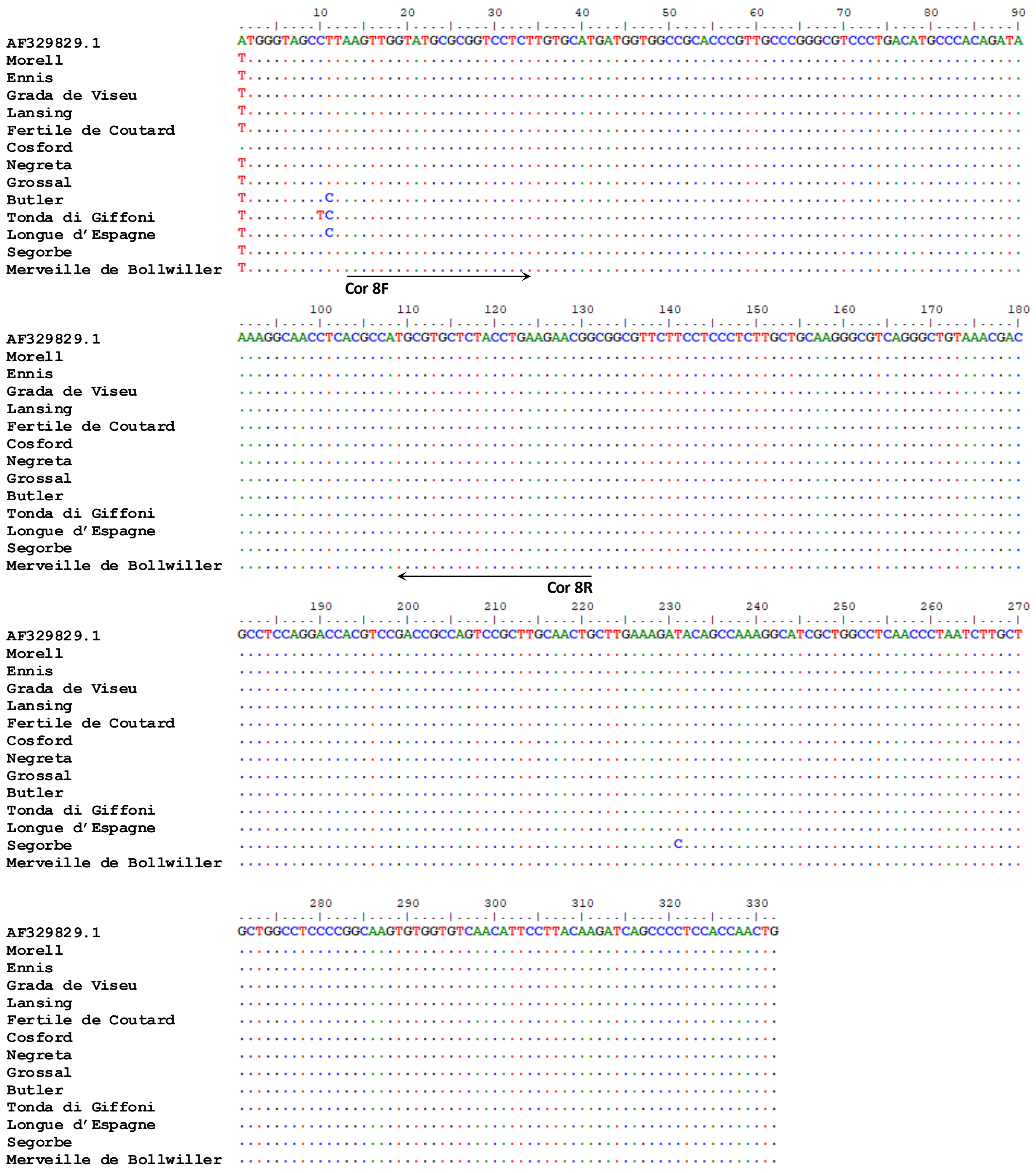

Fig. S2. Sequencing alignments of Cor a 8 gene for all tested hazelnut varieties cultivars. Primers Cor 8F/Cor 8R delimit the DNA region amplified by real-time PCR assays.

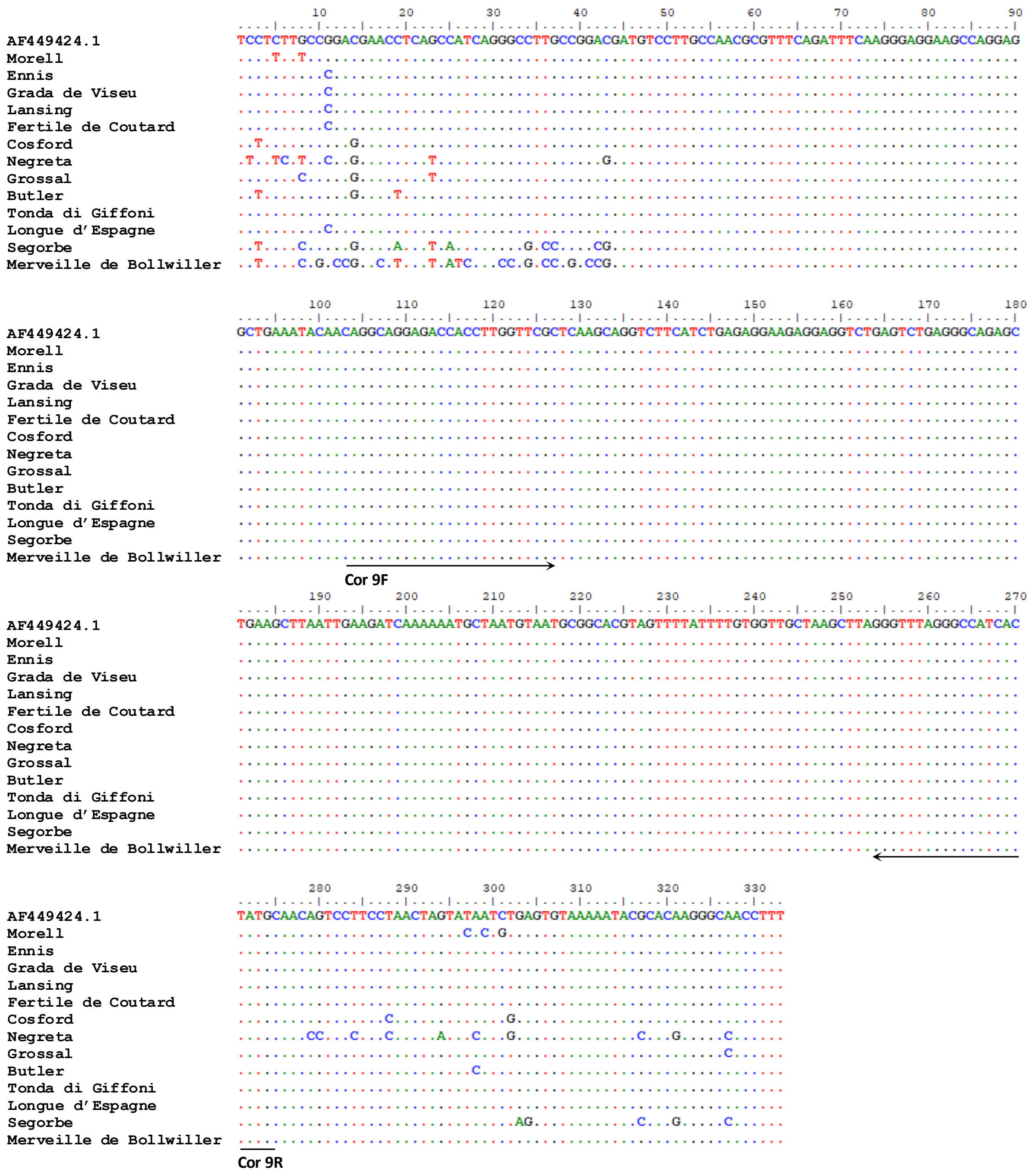

Fig. S3. Sequencing alignments of Cor a 9 gene for all tested hazelnut varieties cultivars. Primers Cor 9F/Cor 9R delimit the DNA region amplified by real-time PCR assays.

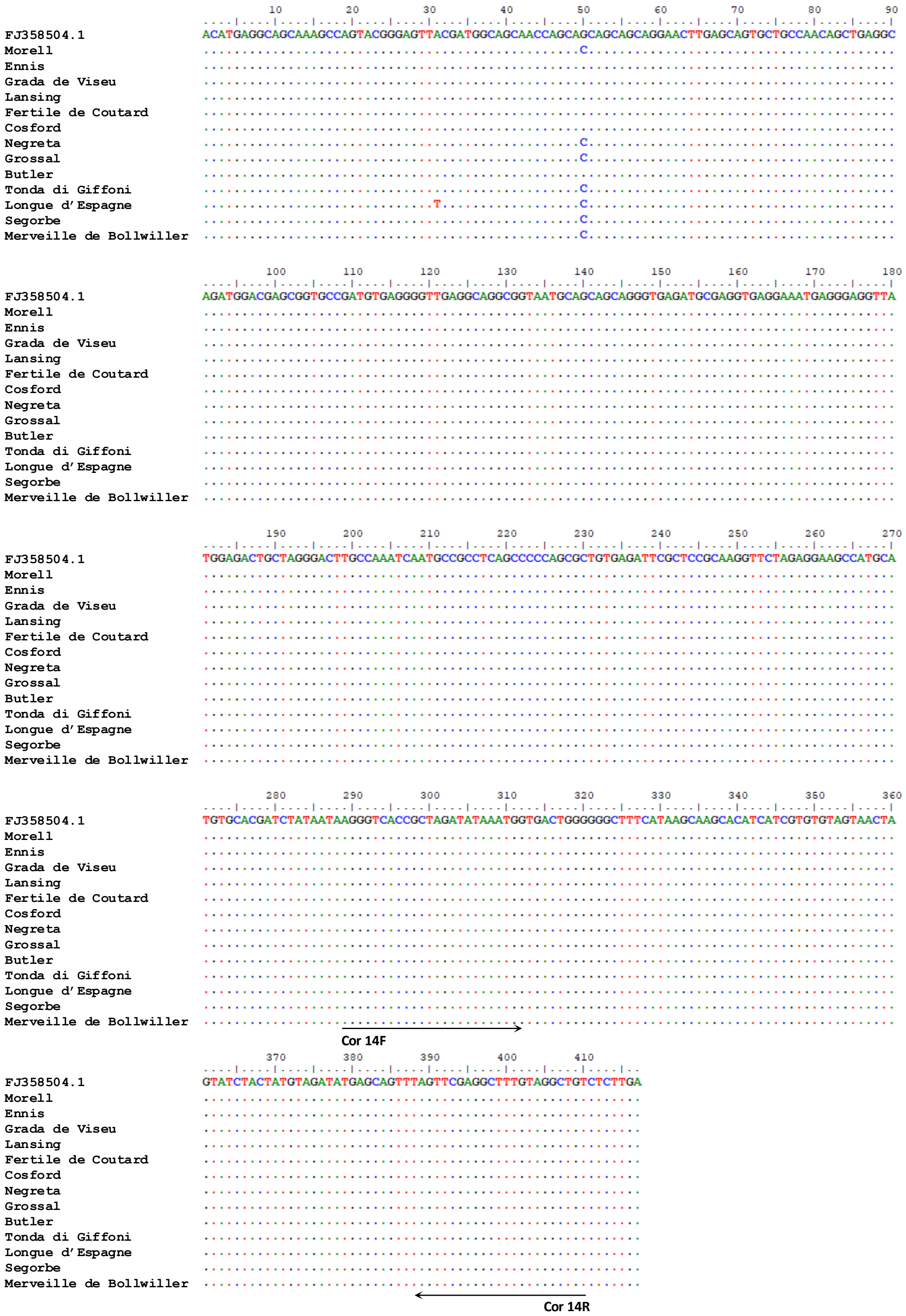

Fig. S4. Sequencing alignments of Cor a 14 gene for all tested hazelnut varieties cultivars. Primers Cor 14F/Cor 14R delimit the DNA region amplified by real-time PCR assays.

Table S2. Reference mobility of each band detected for band pattern matching using CLIQS 1D Pro software (TotalLab, UK).

Zone	Ref Band	Butler	Cosford	Ennis	Fertile de Coutard	Grada de Viseu	Grossal	Longue d'Espagne	Lansing	Merveille de Bollwiller	Morell	Negreta	Segorbe	Tonda di Giffoni
		Reference mobility												
	**1**	0.054				0.047	0.061				0.062			
	**2**	0.072	0.066	0.068		0.071	0.077		0.071					
	**3**				0.084			0.088	0.081					
**A**	**4**		0.134	0.136		0.131	0.133		0.136	0.134	0.135	0.134	0.137	0.135
	**5**					0.181			0.183					
	**6**					0.276				0.281				
	**7**		0.291	0.292		0.29			0.291	0.291	0.289			
	**8**									0.31	0.308			
	**9**	0.4	0.399	0.401	0.402	0.399	0.397	0.402	0.404	0.401	0.4	0.403	0.4	0.397
	**10**	0.433		0.436		0.441			0.437		0.438	0.437	0.436	0.431
	**11**		0.443	0.444					0.447	0.445				
**B**	**12**	0.464	0.458	0.46		0.459	0.461		0.461	0.46		0.459	0.46	0.457
	**13**		0.474	0.472	0.47	0.472		0.467	0.476	0.474	0.467	0.474	0.473	0.47
	**14**	0.482	0.481	0.483	0.486	0.499	0.479	0.485	0.485	0.485	0.483	0.499	0.481	0.479
	**15**	0.495	0.496	0.499	0.501	0.648	0.501	0.501	0.498	0.5	0.5	0.546	0.496	0.494
	**16**							0.525						
	**17**	0.553	0.551	0.553		0.551			0.549	0.554	0.553	0.582	0.547	0.547
	**18**						0.564				0.564			
	**19**	0.582	0.582	0.587	0.577	0.583	0.58	0.576	0.591	0.59	0.597	0.649	0.582	0.582
	**20**				0.617			0.614	0.617			0.612		
	**21**							0.639						
**C**	**22**	0.646	0.648	0.65	0.645	0.673	0.649	0.651	0.651	0.654	0.65	0.674	0.646	0.641
	**23**												0.656	
	**24**	0.67	0.672	0.67	0.668	0.948	0.673	0.673	0.675	0.675	0.674	0.688	0.675	0.67
	**25**								0.689	0.689	0.688	0.691	0.686	0.685
	**26**			0.736			0.737				0.735		0.735	
	**27**			0.744			0.745	0.743	0.745		0.745		0.743	
	**28**				0.763									

**29**				0.774		0.773	0.768			0.767	0.772	0.768	0.773
**30**			0.787	0.79		0.79	0.79	0.791	0.788	0.787	0.788	0.787	0.787
**31**			0.82	0.825	0.823				0.819	0.823			
**32**	0.832	0.833	0.837			0.84	0.833	0.838	0.841	0.838	0.838	0.832	0.83
**33**			0.952		0.976			0.953	0.955	0.954	0.961	0.956	0.957
**34**			0.97	0.968		0.964	0.968	0.97	0.972	0.966	0.974	0.972	0.971
**35**	0.977	0.977	0.984										

Table S3. Frequencies of each band detected in hazelnut electrophoretic profile.

Zone	Band	N	%
A	1	3	23.1
	2	5	38.5
	3	4	30.8
	4	10	76.9
	5	2	15.4
	6	2	15.4
	7	6	46.2
	8	2	15.4
B	9	13	100.0
	10	8	61.5
	11	4	30.8
	12	10	76.9
	13	11	84.6
	14	13	100.0
	15	13	100.0
C	16	1	7.7
	17	10	76.9
	18	2	15.4
	19	13	100.0
	20	4	30.8
	21	1	7.7
	22	13	100.0
	23	1	7.7
	24	13	100.0
	25	6	46.2
	26	4	30.8
	27	6	46.2
	28	1	7.7
	29	7	53.8
	30	10	76.9
	31	5	38.5
	32	11	84.6
	33	8	61.5
	34	10	76.9
	35	3	23.1

Table S4. Similarity matrix of the studied hazelnut varieties based on storage proteins polymorphism.

	Butler	Cosford	Ennis	Fertile de Coutard	Grada de Viseu	Grossal	Longue d'Espagne	Lansing	Merveille de Bollwiller	Morell	Negreta	Segorbe	Tonda di Giffoni
**Cosford**	0.647												
**Ennis**	0.522	0.682											
**Fertile de Coutard**	0.286	0.318	0.384										
**Grada de Viseu**	0.500	0.600	0.625	0.348									
**Grossal**	0.429	0.391	0.500	0.476	0.308								
**Longue d'Espagne**	0.318	0.348	0.407	0.667	0.269	0.571							
**Lansing**	0.440	0.583	0.731	0.423	0.600	0.481	0.500						
**Merveille de Bollwiller**	0.375	0.591	0.680	0.417	0.609	0.423	0.385	0.654					
**Morell**	0.385	0.407	0.667	0.423	0.481	0.600	0.444	0.586	0.654				
**Negreta**	0.476	0.500	0.600	0.524	0.522	0.522	0.545	0.708	0.652	0.640			
**Segorbe**	0.435	0.458	0.680	0.417	0.480	0.609	0.500	0.654	0.600	0.720	0.810		
**Tonda di Giffoni**	0.500	0.524	0.625	0.476	0.545	0.545	0.500	0.667	0.682	0.667	0.944	0.850	
